# Effect of pretreatment with a P2Y_12_ inhibitor in patients with non-ST-elevation acute coronary syndrome: a systematic review and network meta-analysis

**DOI:** 10.3389/fcvm.2023.1191777

**Published:** 2023-07-19

**Authors:** Yachao Li, Mengjie Lei, Zhigang Zhao, Yanli Yang, Lei An, Jingyao Wang, Xue Sun, Cairong Li, Zengming Xue

**Affiliations:** Department of Cardiology, Langfang People’s Hospital, Hebei Medical University, Langfang Core Laboratory of Precision Treatment of Coronary Artery Disease, Langfang, China

**Keywords:** non-ST-segment elevation acute coronary syndrome, antiplatelet, P2Y_12_ inhibitor, pretreatment, meta-analysis

## Abstract

**Background:**

This study aimed to systematically evaluate the effects of different types and doses of pretreatment with P2Y_12_ inhibitors in patients with non-ST-elevation acute coronary syndrome (NSTE-ACS) undergoing percutaneous coronary intervention (PCI).

**Methods:**

Electronic databases were searched for studies comparing pretreatment with different types and doses of P2Y_12_ inhibitors or comparison between P2Y_12_ inhibitor pretreatment and nonpretreatment. Electronic databases included the Cochrane Library, PubMed, EMBASE, and Web of Science. Literature was obtained from the establishment of each database until June 2022. The patients included in the study had pretreatment with P2Y_12_ inhibitors with long-term oral or loading doses, or conventional aspirin treatment (non-pretreatment). The primary endpoint was major adverse cardiac and cerebrovascular events (MACCEs) during follow-up within 30 days after PCI, which included determining the composite endpoints of cardiac death, myocardial infarction, ischemia-driven revascularization, and stroke. The safety endpoint was a major bleeding event.

**Results:**

A total of 119,014 patients from 21 studies were enrolled, including 13 RCTs and eight observational studies. A total of six types of interventions were included—nonpretreatment (placebo), clopidogrel pretreatment, ticagrelor pretreatment, prasugrel pretreatment, double loading pretreatment (double loading dose of clopidogrel, ticagrelor, prasugrel) and P2Y_12_ inhibitors pretreatment (the included studies did not distinguish the types of P2Y_12_ inhibitors, including clopidogrel, ticagrelor, and prasugrel). The network meta-analysis results showed that compared to patients without pretreatment, patients receiving clopidogrel pretreatment (*RR *= 0.78, *95% CI*:0.66, 0.91, *P *< 0.05) and double-loading pretreatment (*RR *= 0.62, *95% CI*:0.41, 0.95, *P *< 0.05) had a lower incidence of MACCEs. There was no statistically significant difference in the incidence of major bleeding events among the six pretreatments (*P* > 0.05).

**Conclusions:**

In patients with NSTE-ACS, pretreatment with P2Y_12_ inhibitors before percutaneous intervention reduced the incidence of recurrent ischemic events without increasing the risk of major bleeding after PCI compared with nonpretreatment. Clopidogrel or double loading dose P2Y_12_ inhibitors can be considered for the selection of pretreatment drugs.

## Introduction

In patients with non-ST-segment elevation acute coronary syndrome (NSTE-ACS), undergoing percutaneous coronary intervention (PCI), platelets are activated during the perioperative period, increasing the risk of stent thrombosis and coronary no-reflow (1). Early oral antiplatelet loading doses can improve prognosis with similar safety (2). In these patients, dual antiplatelet therapy with aspirin and P2Y_12_ inhibitors after PCI was effective in preventing the recurrence of thrombosis and ischemic events (3), also reducing the occurrence of major adverse cardiovascular events (MACEs) (4, 5). However, whether patients with NSTE-ACS should be pretreated with oral P2Y_12_ inhibitors before PCI remains controversial. With the widespread clinical application of new P2Y_12_ inhibitors, data from 20 years ago supported the use of pretreatment (4, 5). Therefore, the long-term efficacy of pretreatment with these drugs remains uncertain (6, 7). The 2020 European Society of Cardiology (ESC) guidelines recommend that pretreatment with a P2Y_12_ receptor inhibitor should be considered in patients with NSTE-ACS who do not plan to undergo an early invasive strategy and do not have a high bleeding risk (HBR) (IIb, C). Routine pretreatment with a P2Y_12_ receptor inhibitor is not recommended in patients whose coronary anatomy is unknown, and early invasive management is planned (III, A). The effects of pretreatment with P2Y_12_ inhibitors on bleeding and recurrent ischemic events in patients with NSTE-ACS undergoing PCI remain controversial. Therefore, we performed a network meta-analysis to assess the efficacy and safety of these therapies.

## Methods

### Inclusion and exclusion criteria

Studies were included for the following reasons: (1) randomized controlled trials and observational studies that compared P2Y_12_ inhibitor pretreatment (intervention group or control group) in patients with NSTE-ACS following PCI; (2) patients were clearly diagnosed with NSTE-ACS (8); and (3) patients were administered oral P2Y_12_ inhibitors before PCI. Additionally, the other treatments and nursing measures in the control group were the same as those in the intervention group and included conventional aspirin treatment.

Studies were excluded for the following reasons: (1) patients had other diseases that affected platelets and blood coagulation, such as severe hematological diseases or immune system diseases; (2) there was a combination of pretreatment and other interventions; and (3) the studies were duplicates.

### Outcomes, definitions, and follow-up

The prognostic indicators synthesized in this study were the results of follow-up 30 days after PCI in patients with NSTE-ACS. The clinical endpoints analyzed included the following: The primary endpoint was major adverse cardiovascular and cerebrovascular event (MACCE) during follow-up within 30 days after PCI, and these MACCEs included the composite endpoints of cardiac death, myocardial infarction, ischemia-driven revascularization, and stroke. The MACCEs in this study were reported in a single study and were not recalculated. The safety endpoint was a major bleeding event.

### Data sources and search strategy

Electronic databases were searched for studies related to pretreatment with P2Y_12_ inhibitors before PCI in patients with NSTE-ACS. The databases included the Cochrane Library, PubMed, EMBASE, and Web of Science. Literature retrieval, literature screening, data extraction, and data analysis were performed in accordance with the Cochrane Collaboration and the Preferred Reporting Items for Systematic Reviews and Meta-analyses (PRISMA) guidelines. Literatures were obtained from the establishment of each database until June 2022.

The search strategy was determined after multiple pre-searches using a combination of MeSH terms and free words, and fine adjustments were made based on a specific database. To reduce publication bias, manual queries were performed for relevant conference papers, academic reports, and dissertations to supplement the search results.

The search terms were as follows: “coronary artery disease”, “acute coronary syndromes”, “ACS”, “non-ST-elevation”, “NSTEMI”, “NSTE-ACS”, “percutaneous coronary intervention”, “PCI”, “angioplasty”, “balloon”, “coronary revascularization”, “high-dose”, “double-dose”, “loading dose”, “pretreatment”, “intensive”, “reload”, “load”, “preload”, “upstream”, “timing”, “antiplatelet”, “ticagrelor”, “clopidogrel”, “prasugrel”, “cangrelor”, and “elinogrel”.

The search strategy was as follows: (“coronary artery disease” OR “acute coronary syndromes” OR “ACS” OR “non-ST-elevation” OR “NSTEMI” OR “NSTE-ACS”) AND (“percutaneous coronary intervention” OR “PCI” OR “angioplasty” OR “balloon” OR “coronary revascularization”) AND (“high-dose” OR “double-dose” OR “loading dose” OR “pretreatment” OR “intensive” OR “reload” OR “load” OR “preload” OR “upstream” OR “timing”) AND (“antiplatelet” OR “ticagrelor” OR “clopidogrel” OR “prasugrel” OR “cangrelor” OR “elinogrel”).

Duplicate literatures were removed using the Endnote software. Literatures were excluded by reading the title and abstract, based on the inclusion and exclusion criteria. Finally, related literatures were excluded by reading the full text. The selected studies were independently checked and cross-checked by two researchers. If the opinions of the two researchers were inconsistent, a third researcher negotiated to reach a consensus.

### Data extraction and assessment of quality

Data extraction and quality evaluation were independently completed by two researchers using the quality evaluation criteria, and a cross-check was performed. If any disagreement occurred about the inclusion of certain data, a third researcher was included to conduct the discussion and reach a consensus. The data extracted from the articles included basic information, inclusion and exclusion criteria, type of research, sample size, type and dosage of P2Y_12_ inhibitors, and outcome indicators. Quality was independently evaluated according to the method recommended in the Cochrane Handbook 5.1.0 (9). The evaluation included seven areas of the randomization method—allocation concealment, blindness of the participants and interveners, blindness of the measurement process, completeness of the outcome indicators (missing data), selective reporting, and other biases. The risk of bias for each area was judged as low, high, or unclear. Grade A included studies that fully met the abovementioned standards and had the lowest possibility of various deviations. Studies that partially met the above-mentioned criteria and had a moderate probability of bias were classified as grade B. Studies with a high probability of bias that did not meet the abovementioned criteria were grade C. The risk of bias in nonrandom studies of interventions (ROBINS-1) was used to evaluate the included cohort studies (10).

### Statistical analysis

Stata software (version 17.0) was used for the data meta-analysis. Descriptive analyses were used for data that could not be quantitatively synthesized. Relative risks (*RRs*) and 95% confidence intervals (*95% CIs*) were calculated for categorical variables. In the presence of a closed loop, the consistency between the direct and indirect comparisons was judged using the node-splitting method. When *P *< 0.05, the inconsistency was evident. The area under the cumulative ranking (SUCRA) showed the possibility of each intervention being the best.

## Results

### Search results

**Figure 1:**
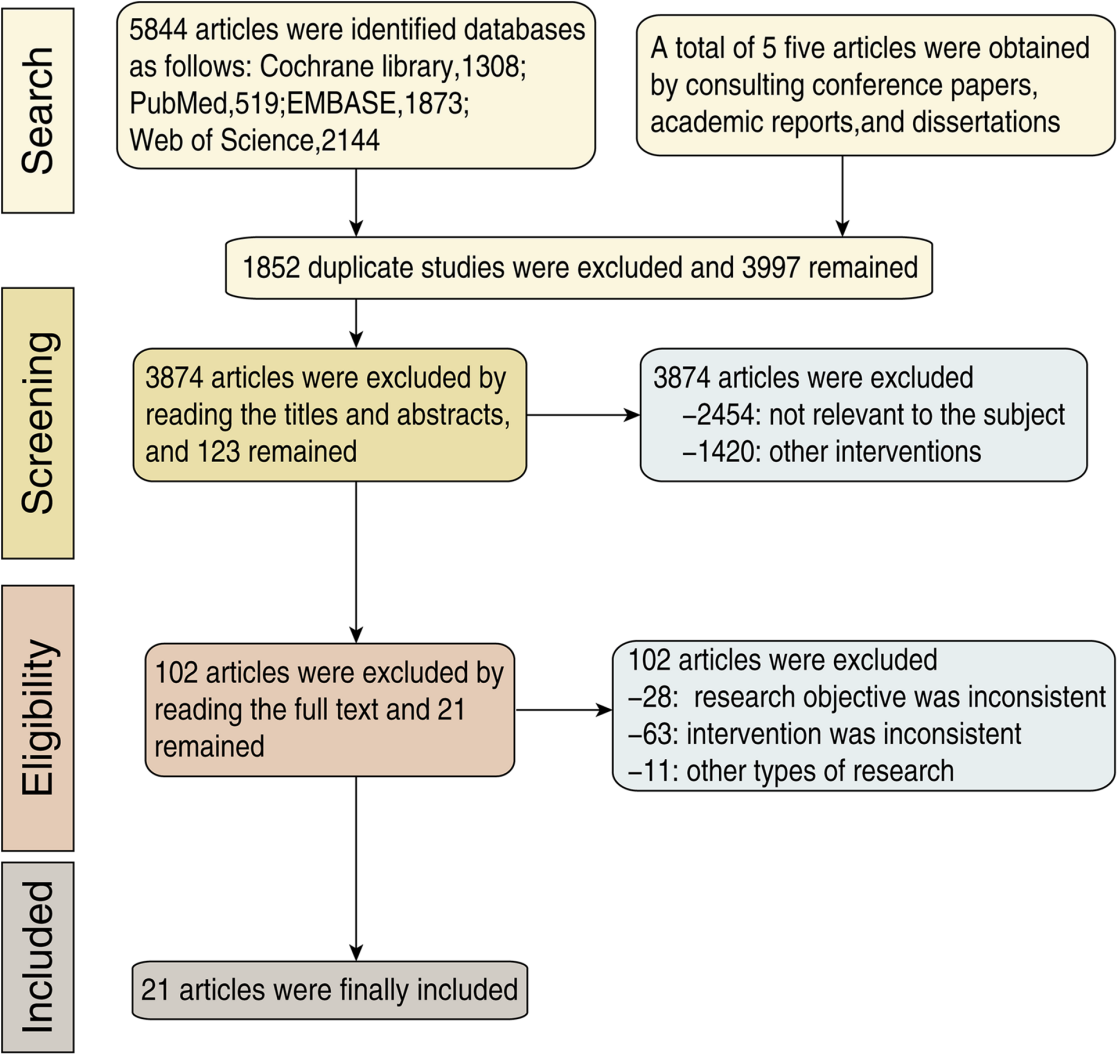
Study selection.

A total of 5,844 articles were retrieved from the databases. Five related articles were obtained through manual retrieval of conference papers, academic reports, and dissertations. Duplicate articles were eliminated using the EndNote software, and 3,997 articles were obtained. On careful assessment of the titles and abstracts, a total of 3,874 articles which were not related to our topic were eliminated. The remaining number of articles was 123. Studies that did not meet the inclusion or exclusion criteria, or had unclear expressions were further eliminated by reading the full text, and 21 articles were obtained (4, 5, 11–29), including 13 RCTs (4, 5, 12, 14, 16, 18–21, 23–26) and eight observational studies (11, 13, 15, 17, 22, 27–29). Literature retrieval and screening processes were carried out in accordance with the PRISMA guidelines; the specific process of screening is shown in Figure 1. Six types of interventions were included—nonpretreatment (placebo), clopidogrel pretreatment, ticagrelor pretreatment, prasugrel pretreatment, double loading pretreatment, and P2Y_12_ pretreatment. Nonpretreatment refers to patients receiving only aspirin treatment before PCI without P2Y_12_ inhibitor treatment. Clopidogrel pretreatment refers to patients receiving clopidogrel (300 mg loading dose) treatment before PCI. Ticagrelor pretreatment refers to patients receiving ticagrelor (180 mg loading dose) before PCI. Prasugrel pre-treatment refers to patients receiving prasugrel treatment (30 mg prasugrel loading dose) before PCI. Double-loading pretreatment refers to treatment with a double-loading P2Y_12_ inhibitor (600 mg clopidogrel loading dose, 360 mg ticagrelor loading dose, and 60 mg prasugrel loading dose) before PCI. P2Y_12_ pretreatment refers to patients included in the original study who received P2Y_12_ inhibitor (clopidogrel, ticagrelor, or prasugrel) treatment before PCI, without distinguishing the types of P2Y_12_ inhibitors.

### General features and methodological quality of the included studies

Twenty-one studies with 119,014 patients with NSTE-ACS were included in this study (4, 5, 11–29). The general characteristics of the included studies are presented in Table 1. The methodological qualities of the included studies are presented in Table 2.

**Table 1:** Basic information of the included studies.

Number	Study	Year	Type	Sample size (intervention/control)	Intervention	Control	MACCE	Bleeding	Follow-up
1	CURE (4)	2001	RCT	6,259/6,303	300 mg LD then 75 mg MD for 3–12 months	No LD; If PCI: 75 mg for 4 weeks	CV death, MI, stroke	TIMI bleeding: major or minor	28 days, 1 year
2	Assali et al. (11)	2001	Observational	235/64	75 mg MD within 5 days or 300 mg LD in morning	300 mg LD immediately after PCI	CV death, MI, UTVR	Major bleeding	In hospital
3	PCI-CURE (5)	2001	RCT	1,313/1,345	300 mg LD then 75 mg MD for 3–12 months	No LD then 75 mg for 4 weeks	CV death, MI,UTVR	TIMI bleeding: major, minor, transfusion	30 days, 1 year
4	CREDO (12)	2002	RCT	900/915	300 mg LD 3–24 h pre-PCI then long term MD	No pretreatment 28 days clopidogrel	Death, MI, UTVR	TIMI bleeding: major or minor	28 days, 1 year
5	Chan et al. (13)	2003	Observational	4,477/332	300 mg pre-PCI	300 mg LD immediately after PCI	Death, MI, UTVR	TIMI bleedings: major or minor	30 days, 6 months, 1 year
6	Thomas Cuisset et al. (14)	2006	RCT	146/146	600 mg clopidogrel LD	300 mg clopidogrel LD	CV death, stent thrombosis, MI, stroke	No	1 month
7	Szük et al. (15)	2007	Observational	1,481/2,679	300 mg clopidogrel LD 6–24 h before PCI	300 mg clopidogrel LD after PCI	all-cause death, MI, UTVR	TIMI major bleedings	30 days
8	Gerald Yong et al. (16)	2009	RCT	132/124	600 mg clopidogrel LD before PCI	300 mg clopidogrel LD before PCI	Death, nonfatal MI, nonfatal stroke, hospitalizations	TIMI bleeding: major or minor	1 month, 6 months
9	Feldman et al. (17)	2010	Observational	467/574	75 mg clopidogrel MD for ≥5 days or 300 mg clopidogrel LD for ≥12 h or 600 mg clopidogrel LD for ≥2 h	600 mg clopidogrel LD for <2 h or 600 mg clopidogrel LD in laboratory	Death, MI,UTVR, stroke	Any bleeding: major(≥4 g/dl Hb) or minor(2–4 g/dl Hb)	In hospital, 1 year
10	ARMYDA5 PRELOAD (18)	2010	RCT	204/205	600 mg clopidogrel LD (4–8 h before PCI)	600 mg clopidogrel LD in laboratory	CV death, MI, or UTVR	TIMI bleeding: major or minor	30 days
11	EARLY ACS (19)	2011	RCT	6,895/2,271	300–600 mg clopidogrel (300 mg early LD, 600 mg LD during PCI)	No-upstream clopidogrel use	death, MI, recurrent ischaemia	TIMI major bleedings, GUSTO severe bleeding/transfusion	In hospital, 30 days
12	Giuseppe Patti et al. (20)	2013	RCT	122/120	600 mg clopidogrel LD	Placebo	death, MI, TVR	Bleeding	30 days
13	ACCOAST (21)	2013	RCT	2,037/1,996	Prasugrel 30 mg pre-PCI then 30 mg after angiogram or before PCI	No pretreatment Prasugrel 60 mg after angiogram	CV death, MI,UTVR, stroke	TIMI bleeding: major or minor	7 days, 30 days
14	ARIAM-Andalucia (22)	2014	Observational	2,797/775	300/600/75 mg clopidogrel	Clopidogrel in laboratory, before (<6 h), or during PCI	CV death, and non fatal reinfarction or stroke/TIA	TIMI bleeding: major, minor or minimal	In hospital
15	Bonello et al. (23)	2015	RCT	106/107	Ticagrelor (180 mg LD, 90 mg twice daily)	Prasugrel (60 mg LD, 10 or 5 mg daily)	CV death, MI,UTVR, stroke	TIMI major bleedings	30 days
16	Hui-Liang Liu et al. (24)	2017	RCT	129/133	Pretreatment:360 mg ticagrelor LD	Pretreatment:180 mg ticagrelor LD	Death, AMI, stroke, and TVR	BARC type 1,2,3a,3b,4,5a,5b bleeding	1 month
17	DUBIUS (25)	2020	RCT	711/721	180 mg ticagrelor LD before PCI	180 mg ticagrelor or 60 mg prasugrel LD after PCI	Death, non fatal MI, or non fatal stroke	BARC type 3, 4 and 5 bleeding	30 days,12 months
18	ISAR-REACT 5 (26)	2020	RCT	1,179/1,186	180 mg ticagrelor before coronary angiography	60 mg prasugrel LD after coronary angiography	Death, MI, or stroke	BARC type 3, 4 and 5 bleeding	30 days, 6 mounths, 12 months
19	Dworeck et al. (27)	2020	Observational	59,894/4,963	Pretreatment:P2Y_12_	No pretreatment	Death, stent thrombosis	In-hospital bleeding	In hospital, 30 days,12 months
20	Pollack et al. (28)	2020	Observational	1,800/1,555	Pretreatment: clopidogrel or prasugrel	Pretreatment: ticagrelor	CV death, MI, stroke	Major bleedings: TIMI, PLATO, BARC	In-hospital, 30 days
21	Leonardo et al. (29)	2022	Observational	735/481	Ticagrelor LD given >6 h before PCI	Ticagrelor LD given <6 h before PCI	CV death,UTVR, stroke/TIA	–	30 days

PCI, percutaneous coronary intervention; CV, cardiovascular; MI, myocardial infarction; UTVR, urgent target vessel revascularization; TVR, target vessel revascularization; MACCEs, major adverse cardiovascular and cerebrovascular events; MD, maintenance dose; LD, loading dose; TIMI, thrombolysis in myocardial infarction; PLATO, Platelet Inhibition and Patient Outcome; BARC, bleeding academic research consortium; GUSTO, global utilization of streptokinase and tissue plasminogen activator.

**Table 2:** Methodological quality evaluation of the included studies.

RCT	Random	Allocation	Blinding	Withdrawn	Selective reporting	Other biases	Grade	
CURE (4)	Low risk	Low risk	Low risk	Low risk	Low risk	Low risk	A	
PCI-CURE (5)	Low risk	Low risk	Low risk	Low risk	Low risk	Low risk	A	
CREDO (12)	Low risk	Low risk	Low risk	Low risk	Low risk	Low risk	A	
Thomas Cuisset et al. (14)	Low risk	High risk	High risk	Low risk	Low risk	Low risk	B	
Gerald Yong et al. (16)	Low risk	Low risk	Low risk	High risk	Low risk	Low risk	B	
ARMYDA5 PRELOAD (18)	Low risk	Low risk	Low risk	Low risk	Low risk	Low risk	A	
EARLY ACS (19)	Low risk	Low risk	Low risk	Low risk	Low risk	Low risk	A	
Giuseppe Patti et al. (20)	Low risk	Low risk	Low risk	Low risk	Low risk	Low risk	A	
ACCOAST (21)	Low risk	Unclear	Low risk	Low risk	Low risk	Low risk	A	
Bonello et al. (23)	Low risk	Unclear	Low risk	Low risk	Low risk	Low risk	A	
Hui-Liang Liu et al. (24)	Low risk	Low risk	Unclear	Low risk	Low risk	Low risk	B	
DUBIUS (25)	Low risk	Low risk	Low risk	Low risk	Low risk	Low risk	A	
ISAR-REACT 5 (26)	Low risk	Low risk	Low risk	Low risk	Low risk	Low risk	A	
Observational	Confounding bias	Selection bias	Intervention classification	Deviations from interventions	Missing outcome data	Measurement	Selective reporting	Overall bias
Assali et al. (11)	Medium risk	Low risk	Low risk	Low risk	High risk	Low risk	Low risk	Medium risk
Chan et al. (13)	Medium risk	Low risk	Low risk	Low risk	Low risk	Low risk	Low risk	Medium risk
Szük et al. (15)	Medium risk	Low risk	Low risk	Low risk	Low risk	Low risk	Low risk	Medium risk
Feldman et al. (17)	Medium risk	Low risk	Low risk	Low risk	Low risk	Low risk	Low risk	Medium risk
ARIAM-Andalucia (22)	Medium risk	Low risk	Low risk	Low risk	Low risk	Low risk	Low risk	Medium risk
Dworeck et al. (27)	Medium risk	Low risk	Low risk	Low risk	Low risk	Low risk	Low risk	Medium risk
Pollack et al. (28)	Medium risk	Low risk	Low risk	Low risk	Low risk	Low risk	Low risk	Medium risk
Leonardo et al. (29)	Medium risk	Low risk	Low risk	Low risk	Low risk	Low risk	Low risk	Medium risk

### MACCEs

#### Meta-analysis results for MACCEs

**Figure 2:**
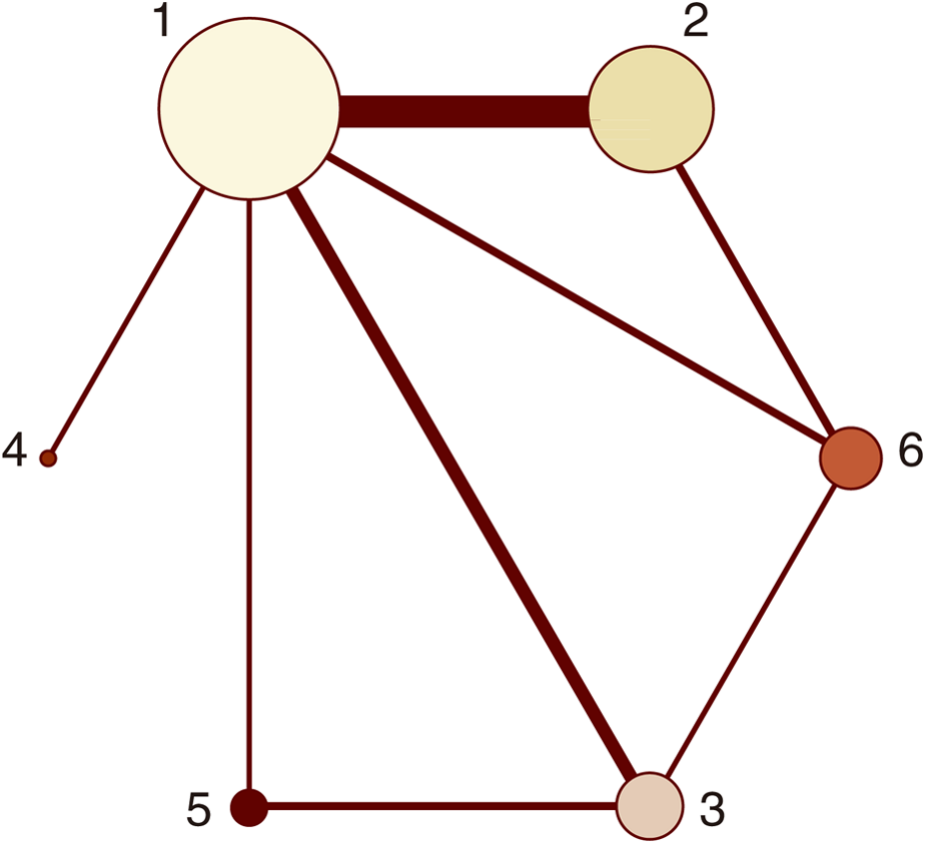
Network plots (MACCEs). 1 = placebo, 2 = clopidogrel pretreatment, 3 = ticagrelor pretreatment, 4 = prasugrel pretreatment, 5 = P2Y_12_ inhibitor pretreatment, 6 = double pretreatment.

**Figure 3:**
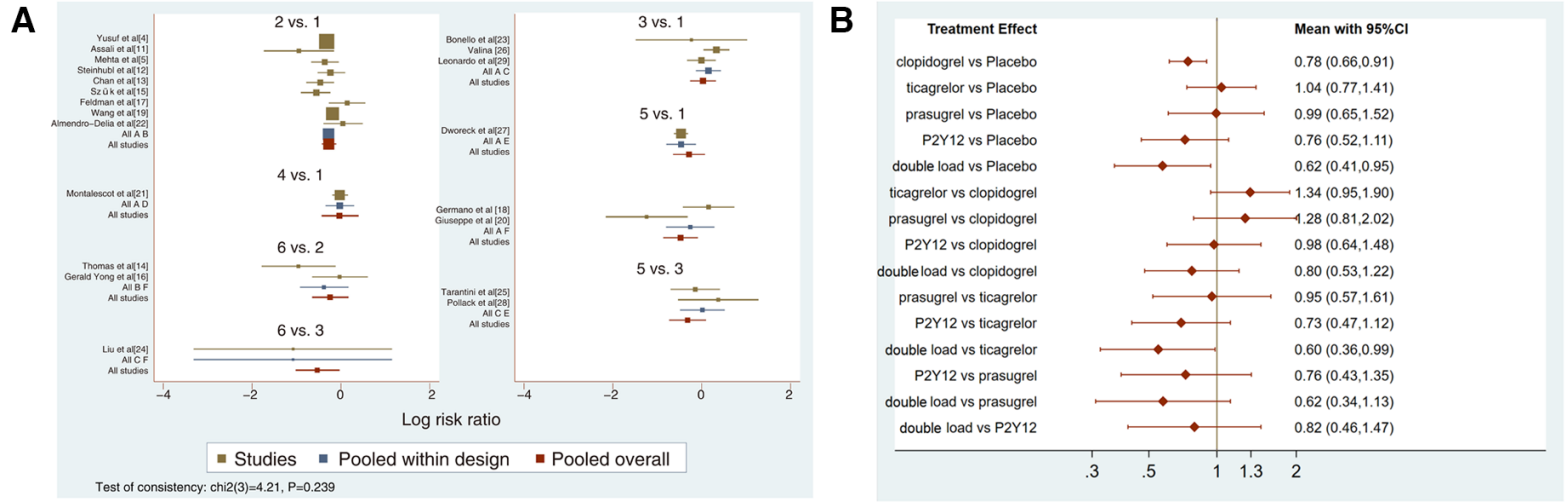
(**A**) direct meta-analysis results (MACCEs). 1 = placebo, 2 = clopidogrel pretreatment, 3 = ticagrelor pretreatment, 4 = prasugrel pretreatment, 5 = P2Y_12_ inhibitor pretreatment, 6 = double pretreatment. (**B**) Network meta-analysis results (MACCEs).

Twenty-one studies with MACCEs were included in the analysis (119,014 patients with NSTE-ACS) (4, 5, 11–29). The results of the inconsistency test showed that the inconsistency was not significant (*I*^2^*^ ^*= 4.21, *P *= 0.239); therefore, the consistency model was used for the analysis. The network plots are shown in Figure 2. The results of the loop inconsistency test showed that loop inconsistency was not significant (*P *> 0.05). The results of the direct meta-analysis are shown in Figure 3A. The network meta-analysis results showed that compared with the patients without pretreatment, the patients receiving clopidogrel pretreatment (*RR *= 0.78, *95% CI*: 0.66, 0.91, *P *< 0.05) and double loading pretreatment (*RR *= 0.62, *95% CI*: 0.41, 0.95, *P *< 0.05) had a lower incidence of MACCEs; compared with the patients who received ticagrelor pretreatment, the patients who received double loading pretreatment (*RR *= 0.60, *95% CI*: 0.36, 0.99, *P *< 0.05) had a lower incidence of MACCEs (Figure 3B).

#### Ranking result of pretreatment for MACCEs

**Figure 4:**
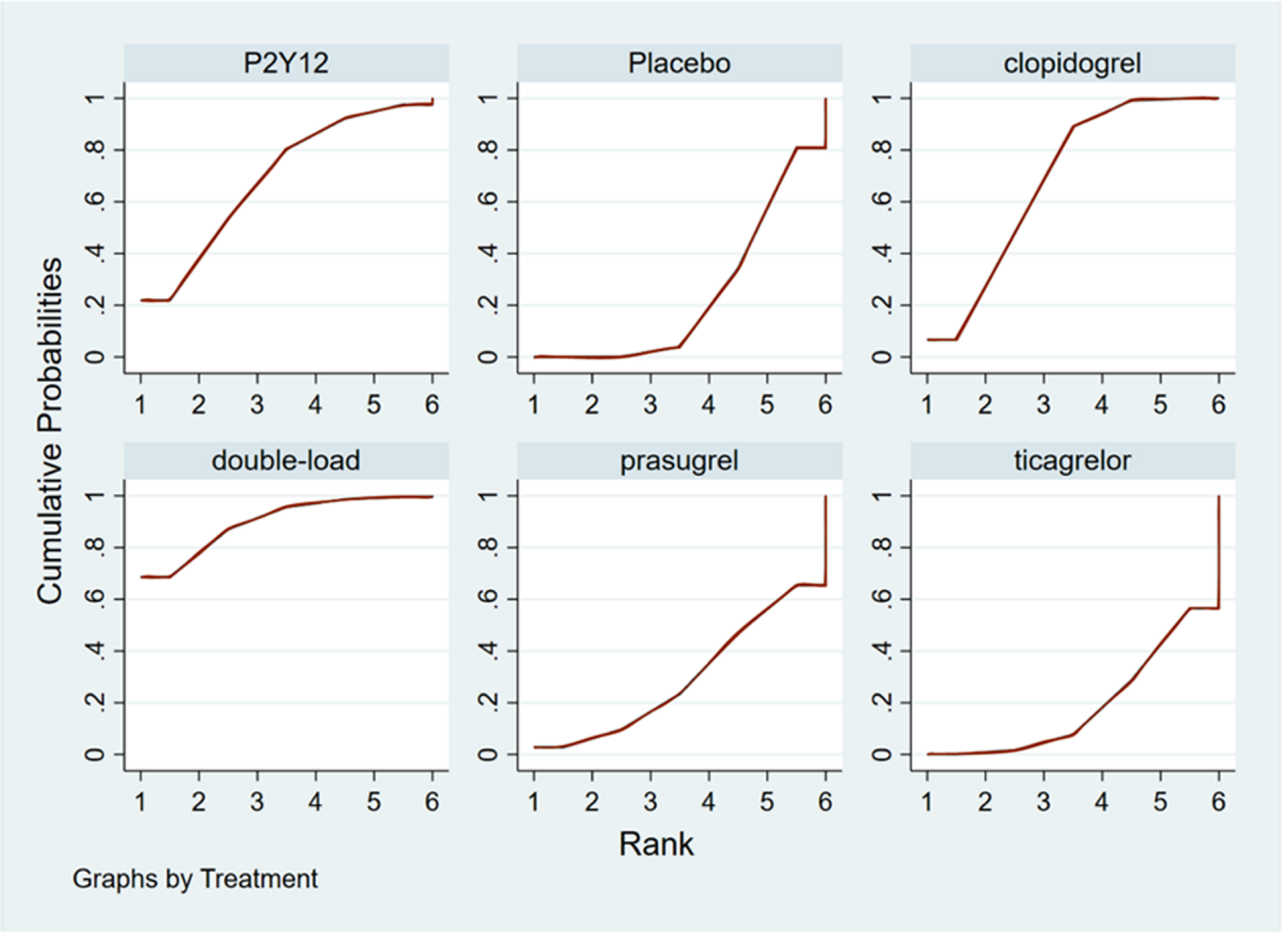
SUCRA curve (MACCEs).

The higher the SUCRA value of the pretreatment method, the fewer the MACCEs that occurred in patients receiving the pretreatment method. The SUCRA values of the six pretreatment methods were ranked from highest to lowest as follows: double-loading pretreatment (SUCRA = 89.9), P2Y_12_ inhibitor pretreatment (SUCRA = 69.1), clopidogrel pretreatment (SUCRA = 68.5), prasugrel pretreatment (SUCRA = 29.7), placebo (SUCRA = 23.8), and ticagrelor pretreatment (SUCRA = 18.9). The SUCRA curves for the different pretreatment methods are shown in Figure 4. Compared to the patients who did not receive pretreatment, those who received clopidogrel pretreatment (*RR *= 0.78, *95% CI*: 0.66, 0.91, *P *< 0.05) and double-loading pretreatment (*RR *= 0.62, *95% CI*: 0.41, 0.95, *P *< 0.05) had a lower incidence of MACCEs. Compared to the patients who received ticagrelor pretreatment, those who received double-loading pretreatment (*RR *= 0.60, *95% CI*: 0.36, 0.99, *P *< 0.05) had a lower incidence of MACCEs (Figure 3B). There were no significant differences in the other pairwise comparisons. Therefore, for MACCEs, it is better for patients to receive clopidogrel pretreatment and double-loading pretreatment than no pretreatment; it is better for patients to receive double-loading pretreatment than ticagrelor pretreatment.

### Major bleeding events

#### Meta-analysis results for major bleeding events

**Figure 5:**
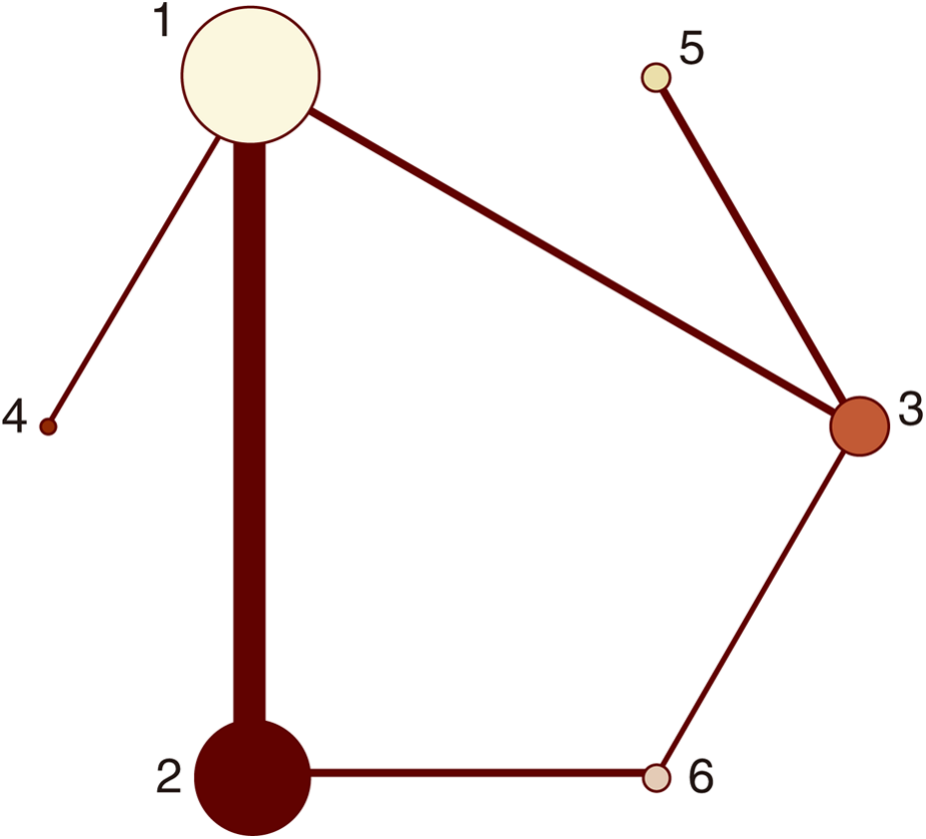
Network plots (major bleeding). 1 = placebo, 2 = clopidogrel pretreatment, 3 = ticagrelor pretreatment, 4 = prasugrel pretreatment, 5 = P2Y_12_ inhibitor pretreatment, 6 = double pretreatment.

**Figure 6:**
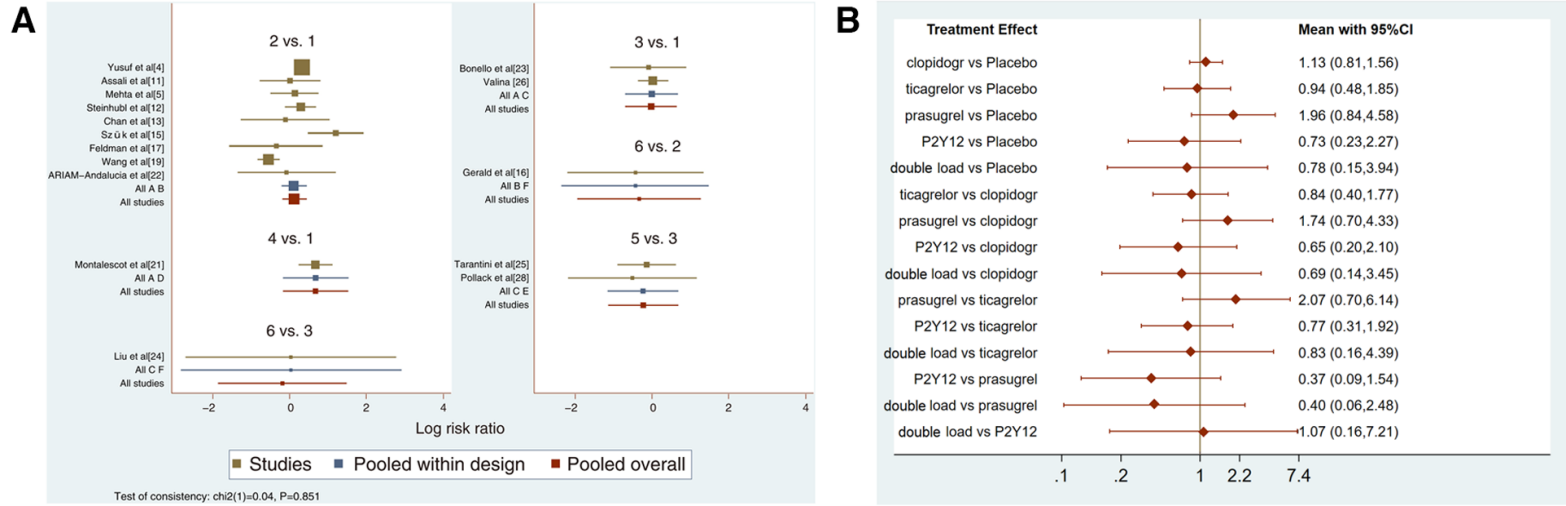
(**A**) direct meta-analysis results (major bleeding). 1 = placebo, 2 = clopidogrel pretreatment, 3 = ticagrelor pretreatment, 4 = prasugrel pretreatment, 5 = P2Y_12_ inhibitor pretreatment, 6 = double pretreatment. (**B**) Network meta-analysis results (major bleeding).

Sixteen studies with major bleeding events were included in the subgroup analysis (52,056 patients with NSTE-ACS) (4, 5, 11–13, 15–17, 19, 21–26, 28). The results of the inconsistency test showed that the inconsistency was not significant (*I^2^* = 0.04, *P *= 0.851); therefore, the consistency model was used for the analysis. The network plots are shown in Figure 5. The results of the loop inconsistency test showed that loop inconsistency was not significant (*P *> 0.05). The results of the direct meta-analysis are shown in Figure 6A. The network meta-analysis results showed no statistically significant differences in the incidence of major bleeding events among the six interventions (Figure 6B).

#### Ranking result of pretreatment for major bleeding events

**Figure 7:**
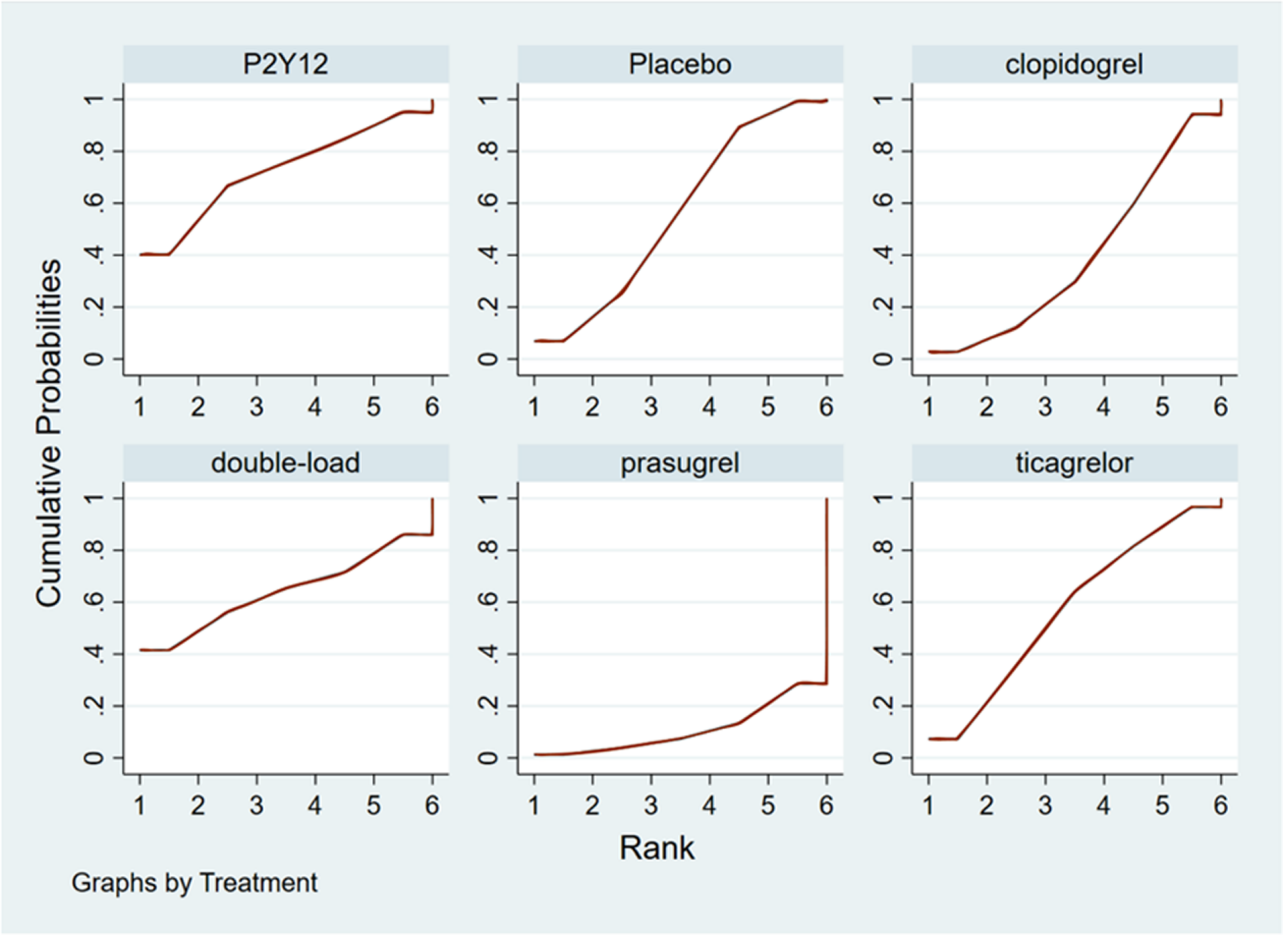
SUCRA curve (major bleeding).

The higher the SUCRA value of the pretreatment method, the fewer major bleeding events occurred in the patients who received the pretreatment method. The SUCRA values of the six pretreatment methods were ranked from highest to lowest as follows: P2Y_12_ inhibitor pretreatment (SUCRA = 72.0), double-loading pretreatment (SUCRA = 63.6), ticagrelor pretreatment (SUCRA = 56.9), placebo (SUCRA = 56.5), clopidogrel pretreatment (SUCRA = 40.4), and prasugrel pretreatment (SUCRA = 10.5). The SUCRA curves for different pretreatment methods are shown in Figure 7. There were no significant differences in the pairwise comparisons.

### Publication bias and contribution plot

**Figure 8:**
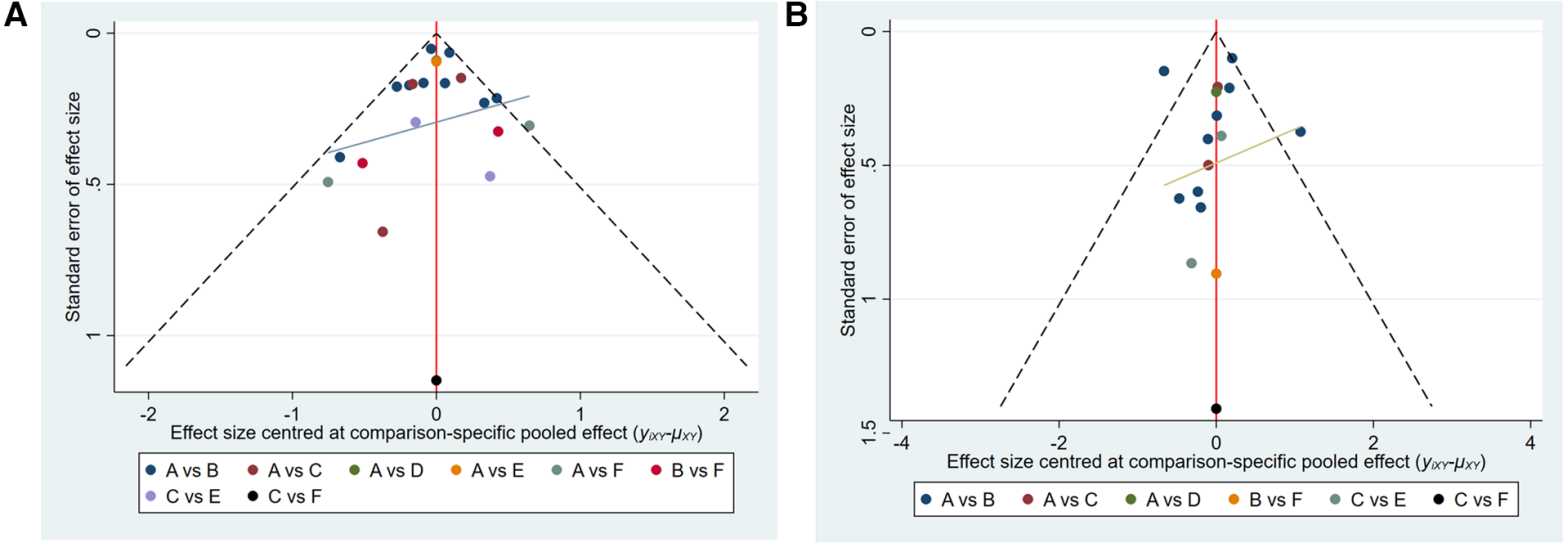
(**A**) publication bias (MACCEs). A = placebo, B = clopidogrel pretreatment, C = ticagrelor pretreatment, D = prasugrel pretreatment, E = P2Y_12_ inhibitor pretreatment, F = double pretreatment. (**B**) Publication bias (major bleeding). A = placebo, B = clopidogrel pretreatment, C = ticagrelor pretreatment, D = prasugrel pretreatment, E = P2Y_12_ inhibitor pretreatment, F = double pretreatment.

**Figure 9:**
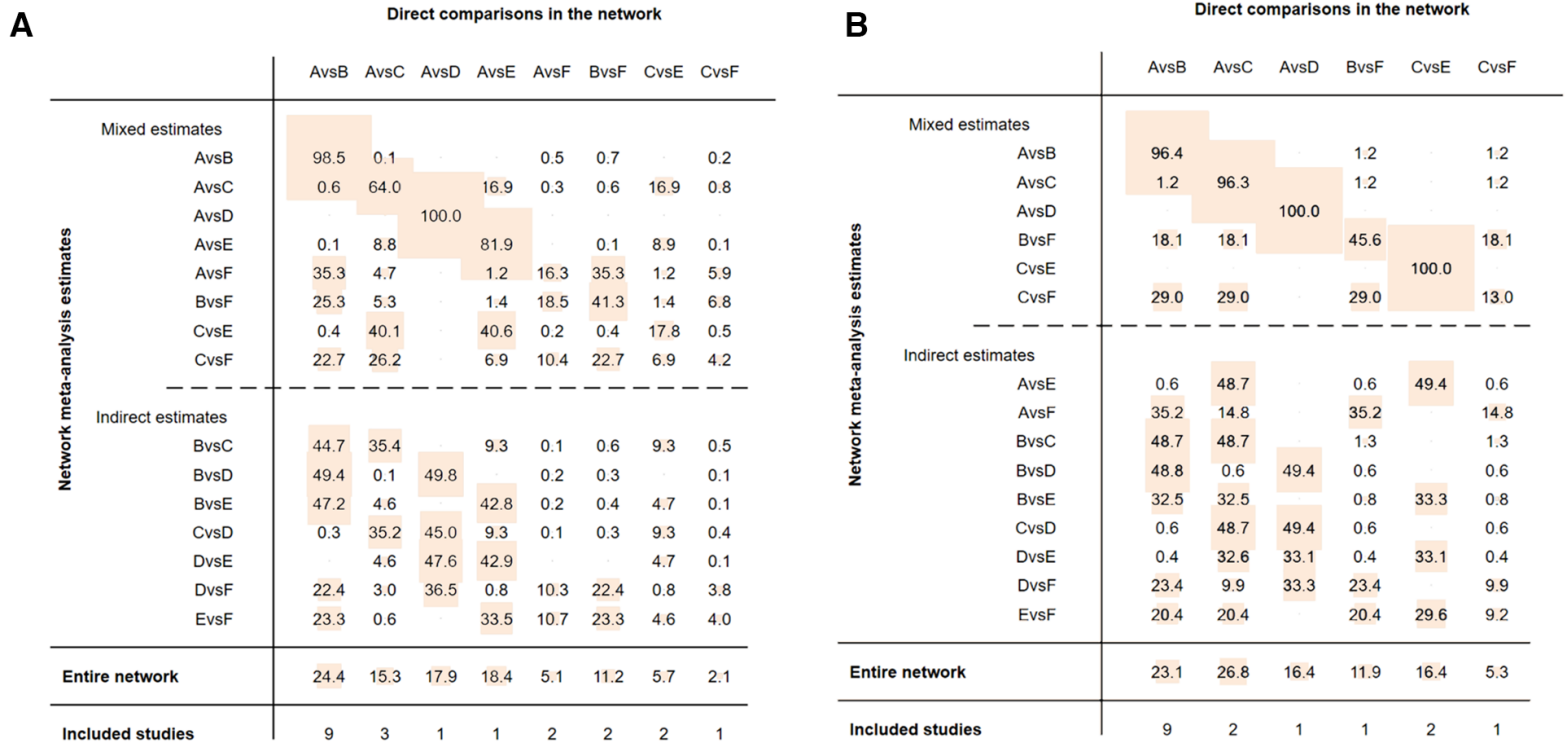
(**A**) contribution plot (MACCEs). Contribution plot for the network. The size of each square is proportional to the weight attached to each direct summary effect (horizontal axis) for the estimation of each network summary effect (vertical axis). The numbers re-express the weights as percentages. (A = placebo, B = clopidogrel pretreatment, C = ticagrelor pretreatment, D = prasugrel pretreatment, E = P2Y_12_ inhibitor pretreatment, F = double pretreatment). (**B**) Contribution plot (major bleeding). Contribution plot for the network. The size of each square is proportional to the weight attached to each direct summary effect (horizontal axis) for the estimation of each network summary effect (vertical axis). The numbers re-express the weights as percentages. (A = placebo, B = clopidogrel pretreatment, C = ticagrelor pretreatment, D = prasugrel pretreatment, E = P2Y_12_ inhibitor pretreatment, F = double pretreatment).

Funnel plots were constructed to test for publication bias (Figures 8A,B). Funnel plots showed that there was a publication bias in this study. A total of 21 studies were included in the meta-analysis. However, the number of studies included was insufficient for each comparison between the two groups. According to the Cochrane Handbook, funnel plots should only be used when the number of studies included is not less than 10 for each outcome indicator. With an extremely small number of included studies, the test efficiency was too low to distinguish between opportunity and true asymmetry. However, two retrieval methods were used to reduce publication bias. Duplicate published studies were excluded to avoid the use of the same data. A contribution plot was constructed for the network (Figures 9A,B).

## Discussion

Twenty-one studies involving 119,014 patients with NSTE-ACS were included in this network meta-analysis. We compared different pretreatment strategies for P2Y_12_ inhibitors to analyze the impact of P2Y_12_ inhibitor pretreatment on the incidence of MACCEs and major bleeding events in patients with NSTE-ACS. The results were as follows: 1) pretreatment with a P2Y_12_ inhibitor before PCI in patients with NSTE-ACS reduced the incidence of MACCEs without increasing the risk of major bleeding events; 2) clopidogrel or a P2Y_12_ inhibitor with a double-loading dose could be selected as the pretreatment scheme; and 3) compared with pretreatment with ticagrelor, a P2Y_12_ inhibitor with a double loading dose could reduce MACCEs.

Current guidelines recommend that patients with ST-elevation myocardial infarction be administered P2Y_12_ inhibitors orally before PCI (30). However, whether P2Y_12_ inhibitor pretreatment should be administered to patients with NSTE-ACS remains controversial (31). In addition, several meta-analyses have discussed P2Y_12_ inhibitor pretreatment (32–37), but there were differences in the types of objects in these meta-analyses. Some studies focused on patients with ACS or all patients receiving PCI (32–35) and did not distinguish between NSTE-ACS patients. Two meta-analyses discussed P2Y_12_ inhibitor pretreatment in patients with NSTE-ACS, and one meta-analysis included patients with NSTE-ACS (36). However, considering that there are many methods for P2Y_12_ inhibitor pretreatment (different drug types and timings), if the methods of P2Y_12_ inhibitor pretreatment are not differentiated, this may lead to some heterogeneity in the meta-analysis. Another network meta-analysis divided the interventions into six groups: early clopidogrel, delayed clopidogrel, early prasugrel, delayed prasugrel, early ticagrelor, and delayed ticagrelor (37). The duration of follow-up was between 1 and 12 months in the nine included studies, which may also lead to some heterogeneity in the meta-analysis. In addition, we believe that the risks and benefits of P2Y_12_ inhibitor pretreatment are mainly shown within 1-month of PCI. Therefore, we systematically reviewed all direct and indirect evidence from randomized controlled trials and observational cohort studies to establish this network meta-analysis, clarify the potential risks and benefits of P2Y_12_ inhibitor pretreatment in patients with NSTE-ACS, and compare the differences between pretreatment schemes. Based on the type, dosage, and timing of P2Y_12_ inhibitors, the interventions were divided into placebo, clopidogrel, ticagrelor, prasugrel, P2Y_12_ inhibitors, and double-loading pretreatment.

We ranked all potential combinations of P2Y_12_ inhibitor pretreatments for MACCEs and bleeding events in patients with NSTE-ACS. For MACCEs, the SUCRA values indicated that the priority of the selected pretreatment scheme was double-loading pretreatment, P2Y_12_ inhibitor pretreatment, clopidogrel pretreatment, prasugrel pretreatment, placebo, and ticagrelor pretreatment. The network meta-analysis results showed that, compared with patients without pretreatment, patients who received clopidogrel pretreatment (*P *< 0.05) and double-loading pretreatment (*P *< 0.05) had a lower incidence of MACCEs. Compared to patients who received ticagrelor pretreatment, those who received double-loading pretreatment (*P *< 0.05) had a lower incidence of MACCEs. For major bleeding events, the SUCRA values suggested that the priority of the selected pretreatment scheme was P2Y_12_ pretreatment, double-loading, ticagretor, placebo, clopidogrel, and prasugrel pretreatment. Therefore, pretreatment with P2Y_12_ inhibitors significantly reduced the number of recurrent ischemic events without increasing the occurrence of major bleeding events. Clopidogrel or P2Y_12_ inhibitors (clopidogrel, ticagrelor and prasugrel) with a double-loading dose should be selected as the pretreatment protocols for patients with NSTE-ACS.

Clopidogrel was the main pretreatment drug in the early studies. Clopidogrel was used as a pretreatment drug in 13 of the 21 studies included in our study. Compared with no pretreatment, the incidence of MACCEs within 30 days of PCI was lower. However, compared to a single loading dose (300 mg) of clopidogrel, a double-loading dose (600 mg) of clopidogrel did not show an advantage. The pretreatment method included a maintenance dose of 75 mg clopidogrel for 5 days or a loading dose of 300 mg clopidogrel before PCI. An early CURE study showed that the incidence of MACEs in patients with NSTE-ACS in the clopidogrel group was significantly reduced (9.3% vs. 11.4%, *P *< 0.001); however, the number of major bleeding events was significantly higher in patients with NSTE-ACS than in those in the control group (3.7% vs. 2.7%, *P *= 0.001) (4). Additionally, there was no significant difference in the number of fatal bleeding events between the two groups (2.1% vs. 1.8%, *RR *= 0.80, *P *= 0.13). Also, there were different research results regarding the timing of pretreatment. The onset of clopidogrel administration was relatively slow. Compared with patients who received clopidogrel pretreatment >6 h before PCI, those who received clopidogrel pretreatment <6 h before PCI had significantly fewer primary ischemic events (14). The pretreatment clopidogrel dose can be doubled in patients requiring rapid platelet inhibition within 6 h (18). With the gradual development and popularization of PCI, the emergence of new and more powerful antiplatelet drugs, and extensive application of drug-eluting stents in clinical practice, research on the application of new P2Y_12_ inhibitors in the pretreatment of patients with NSTE-ACS before PCI has gradually increased. This meta-analysis included eight studies on ticagrelor and prasugrel, but two studies supported double-loading dose P2Y_12_ inhibitors (24, 26), including ticagrelor and prasugrel, which were superior to pretreatment with ticagrelor in terms of the incidence of MACCEs, which may be related to the rapid inhibition of platelet activity. However, there are few studies, and the results may be inconsistent. Similarly, compared with a placebo, the use of a P2Y_12_ inhibitor with a double-loading dose can inhibit platelet activity more quickly and reduce the occurrence of MACCEs.

Our network meta-analysis has increased our understanding of the existing evidence related to P2Y_12_ receptor inhibitor pre-treatment and clarified that P2Y_12_ inhibitor pretreatment can reduce MACCEs within 30 days after PCI without increasing the number of bleeding events. However, more researches are needed to compare different P2Y_12_ receptor inhibitor pretreatment schemes and determine the optimal P2Y_12_ inhibitor pretreatment program for patients with NSTE-ACS. Compared with previous meta-analyses (32–37), our study showed some differences and advantages—this meta-analysis fully considered the differences in drug types, dosage, and administration timing of P2Y_12_ inhibitors to evaluate the different pretreatment schemes; it summarizes all available information, including direct evidence and indirect estimates and provides the potential benefits and risks of each pretreatment method, and the age range of the patients included in the study was wide, ensuring the external validity of the results.

In summary, this study supports the use of pretreatment with P2Y_12_ inhibitors in patients with NSTE-ACS. The suggested timing of pretreatment is when NSTE-ACS is diagnosed or before angiography. The pretreatment protocol is suggested to be clopidogrel [including 75 mg daily maintenance ≥5 days or loading dose (300 mg) before PCI] or a double-loading dose of a P2Y_12_ inhibitor (including 600 mg of clopidogrel, 360 mg of ticagrelor and 60 mg of prasugrel). Additionally, this study found that a double-loading dose of a P2Y_12_ inhibitor was superior to a single-loading dose (180 mg) of ticagrelor. The above-recommended pretreatment method can reduce the incidence of MACCEs without increasing the risk of major bleeding events.

Current guidelines do not recommend routine pretreatment before angiography because of the risk of intracranial hemorrhage and delayed CABG. If the patient needs to undergo coronary artery bypass grafting after angiography, ticagrelor should be discontinued for at least 3 days, clopidogrel for at least 5 days, and prasugrel for at least 1 week (38). Therefore, routine pre-treatment may cause high platelet inhibition and increase the risk of bleeding in some patients during the perioperative period. We observed that prasugrel alone increased the risk of major bleeding. In addition, pretreatment with clopidogrel and ticagrelor did not increase the risk of major bleeding (5, 24). Some patients refused coronary artery bypass therapy after coronary angiography because of socioeconomic factors. These patients require long-term antiplatelet therapy to improve ischemia; therefore, there is no need to discontinue P2Y_12_ inhibitors.

## Limitation

However, this study has some limitations. First, the outcome indicators in this study were based on follow-up 30 days after PCI, which lacked laboratory-related indicators (platelet aggregation) and in-hospital bleeding. Future investigations of the perioperative period and related laboratory indicators should be performed. Second, the 21 studies included in our analysis comprised 13 randomized controlled trials and eight observational studies. In addition, the current research on P2Y_12_ inhibitor pretreatment in patients with NSTE-ACS before PCI mainly included clopidogrel pretreatment. Third, for the definition of some endpoints, there were differences between the studies, especially TIMI bleeding, GUSTO bleeding, and MACCEs, which may lead to heterogeneity in this indicator. Fourth, it included open-label randomized controlled trials because it is difficult to use double-blind P2Y_12_ inhibitor strategies and timing. Finally, the comparison between the use of a P2Y_12_ inhibitor with a double loading dose and ticagrelor included only three studies, with a confidence interval of 0.36–0.99. Therefore, the results of this study need to be interpreted carefully.

## Conclusions

For patients with NSTE-ACS, pretreatment with P2Y_12_ inhibitors before PCI reduced the incidence of recurrent ischemic events without increasing the risk of major bleeding events after PCI compared with nonpretreatment. Clopidogrel or double loading dose P2Y_12_ inhibitors can be considered for the selection of pretreatment drugs.

## Data Availability

The original contributions presented in the study are included in the article/Supplementary Material, further inquiries can be directed to the corresponding author.
